# Selenium Biomarkers in Prostate Cancer Cell Lines and Influence of Selenium on Invasive Potential of PC3 Cells

**DOI:** 10.3389/fonc.2013.00239

**Published:** 2013-09-23

**Authors:** Wouter Hendrickx, Julie Decock, Francis Mulholland, Yongping Bao, Susan Fairweather-Tait

**Affiliations:** ^1^Department of Nutrition, Norwich Medical School, University of East Anglia, Norwich, UK; ^2^School of Biological Sciences, University of East Anglia, Norwich, UK; ^3^Institute of Food Research, Norwich Research Park, Norwich, UK

**Keywords:** selenium, prostate cancer, invasion, biomarkers, GPx, TrxR1

## Abstract

Dietary selenium intake has been linked to reduced cancer risk, however the underlying mechanisms are yet unknown. We question the commonly used practice of applying selenium concentrations found in human blood to *in vitro* studies and evaluated the utility of biomarkers, e.g., glutathione peroxidase 1 (GPx1) and thioredoxin reductase 1 (TrxR1), to determine appropriate selenium levels for *in vitro* work. Furthermore, we investigated the effects of Se-methylselenocysteine (SeMSC) on prostate cancer cell migration and invasion. After excluding cytotoxicity, we demonstrated that prostate cancer cell lines respond differently to selenium treatment as observed through biomarker assessment. We found that the maximum levels of GPx1 activity and TrxR1 expression were reached at lower selenium concentrations in LNCaP compared to PC3 cells, and PC3 compared to DU145 cells. Therefore the use of selenium concentrations extrapolated from human studies for *in vitro* work may be applicable when further informed using a readout of selenium repletion including use of selenium responsive biomarkers. No effect on PC3 migration or invasion was observed after long term SeMSC treatment; however a slight increase was found when treatment was solely administered during the assay. The opposite could be observed when cells were cultured under low serum conditions, with a significant increase in migration upon long term but not upon acute SeMSC treatment. To conclude, these findings indicate that it is imperative to study the selenium sensitivity of an *in vitro* model preferably using biomarkers before investigating any effects on biological processes, or before comparing models.

## Introduction

Selenium is an essential trace element and plays an important role in protection against cardiovascular disease, inflammation and inflammatory disorders, diabetes, infertility, and cancer (1). Selenium regulates protein function through its incorporation into selenoproteins as selenocysteine (1). Most selenoproteins have a functional role that impacts on chronic diseases such as cancer through the management of reactive oxygen species (ROS), present in numerous antioxidant defense systems throughout the body (2).

It has become clear that there is a very narrow margin between selenium deficiency and toxicity, with beneficial levels on health being dependent on the form of selenium, level of exposure, and selenium status (3). Selenium can be measured in whole blood, blood fractions (plasma, serum, red blood cells), hair, nails, and urine. Plasma selenium levels below 0.6 μM (40–50 ng/ml) are considered deficient, and risk of toxicity occurs at levels higher than 2 μM (160 ng/ml), with reports of toxic effects at concentrations higher than 3 μM (250 ng/ml) (4–6). Plasma selenium concentrations are considered to be adequate at 1.1–2 μM (90–160 ng/ml), when selenoprotein P levels reach a plateau and cancer protective benefits may be maximized (7). Daily dietary recommendations have traditionally been based on the quantity of selenium required for optimal glutathione peroxidase 3 (GPx3) or erythrocyte GPx1 activity in blood. As such the dietary reference in the UK is set at 60 μg/day for women and 75 μg/day for men although the mean intakes are 45–55 μg/day (1). In the US intakes are higher than in the UK, whereas in parts of China there are extremes of intake (related to local soil conditions), with both deficient and toxic levels (8).

The aim of the present work was to interrogate the value of extrapolating selenium concentrations found in human blood to *in vitro* studies. The use of biomarkers is now common practice in clinical nutrition studies and sometimes in animal models. However, in *in vitro* models, the amount of selenium used is at best extrapolated from “physiological relevant” serum concentrations. We evaluated the use of biomarkers such as glutathione peroxidase 1 (GPx1) and thioredoxin reductase 1 (TrxR1) as markers for selenium repletion and this within the non-toxic range of selenium treatment. GPx1 is one of the most abundant selenoproteins and its expression is highly sensitive to a fall in selenium supply and oxidative stress (9, 10). In general, enhanced GPx1 function is associated with increased protection against oxidative damage (11). TrxR1 is a highly selenium sensitive selenoprotein (11) and is of interest as a selenium biomarker for prostate cancer cells as it has been shown to respond to selenium treatment past the repletion point of GPx (12).

Further, we investigated the effect of selenium on cancer cell motility using migration and invasion assays of PC3 cells. Cancer cells often have enhanced growth and metastatic potential. To date, only a few studies have investigated the effect of selenium on migration and invasion of cancer cells *in vitro* (13–17). Although there are discrepancies between the findings from these studies, treatment with various selenium compounds has generally resulted in a decrease in the migration and, in some instances, the invasion of cancer cells. As no data have been published on the role of selenium in cell motility of prostate cancer cell lines, the aim of the present work was to determine the effect of Se-methylselenocysteine (SeMSC) on the migration of PC3 cells and their invasion through matrigel.

There are limited comparative data on the toxicity of selenium compounds in cell systems, hence we compared the cytotoxicity of selenomethionine (SeMet), SeMSC, and selenite in three different prostate cancer cell lines (18–23). SeMet represents the major form of selenium in plant crops, while SeMSC can be found in broccoli, garlic, and onions, especially when grown under selenium-rich conditions (24, 25). Sodium selenite is water-soluble and is the most commonly used form in food and vitamin supplements (26).

In the present study the utility of GPx1 and thioredoxin reductase 1 as *in vitro* selenium biomarkers was assessed in three different prostate cancer cell lines and the effects of selenium treatment on migration and invasion of PC3 cells was investigated.

## Materials and Methods

### Cell culture

LNCaP, PC3, and DU145 are cell lines derived from prostate cancer metastasis isolated from the lymph nodes, bone, and brain, respectively. All cell lines were procured from the American Type Culture Collection bank (ATCC) and were maintained in Dulbecco’s Modified Eagle Medium/F12 (DMEM/F12) plus GlutaMAX™ (2.5 mM l-Alanyl-l-Glutamine) with Hyclone-defined fetal bovine serum (FBS, Thermo Scientific) and 1% penicillin–streptomycin (penicillin 5000 units/ml, streptomycin 5000 μg/ml, Gibco). The use of Hyclone-defined FBS containing 380 nM total selenium, according to manufacturer’s batch analysis, resulted in control samples containing 38 nM or 10 nM total selenium under respectively 10% or low 2.5% serum conditions. Low 2.5% serum culture conditions were achieved by gradually reducing the amount of FBS. Selenium treatment of several cell lines with selenite (Sigma-Aldrich), SeMet (Sigma-Aldrich), or SeMSC (Sigma-Aldrich), was conducted for a duration 48 h for short-term (acute exposure) or for 30 days which allows longer term adaptation to occur.

### Protein extraction and quantification

Cells were lysed at 80% confluency in 100 mM Tris lysis buffer pH 7.4 supplemented with 0.1% Triton X-100 and a protease inhibitor cocktail (Roche). Cell pellets (6 × 10^6^ cells) were ground in 350 μl lysis buffer using a microcentrifuge tube pestle (Sample grinding kit, GE Healthcare) and sonicated on ice (Biologics 300V/T with 3.9 mm tip, 40% power, 50% pulse, 7 min). After sonication, samples were centrifuged at 12,000 × *g*, 4°C for 10 min, and stored at −80°C. The protein concentration was quantified using a BCA assay kit (Thermo Scientific) and absorbance was read at 562 nm.

### MTT viability assay

Cells were seeded at 10 × 10^3^ cells/well in a 96-well tissue culture plate. After 24 h, the cells were treated with 10 different concentrations of selenium supplementation for 48 h. The treatment was removed and cells were incubated with MTT (0.45 mg/ml, Sigma) at 37°C for 2 h. Finally cells were incubated with DMSO (Fisher) at room temperature for 5 min. Changes in metabolism were used as an indicator of cell viability which was calculated from absorbance values at 550 and 630 nm (wavelength correction) using the MARS analysis software (BMG Labtech, version 1.11).

### GPx enzyme activity assay

Glutathione peroxidase activity was assessed using an assay mixture of 100 mM Tris, 5 mM EDTA, 1.5 mM sodium azide, 0.1% Triton X-100, 0.25 mM NADPH (Sigma), 3 mM glutathione (Sigma), and 1 unit glutathione reductase (Sigma). Cell extracts were used at 0.5–2 mg/ml according to the enzyme activity of each cell line. The rate of increase in absorbance at 340 nm was monitored at 37°C every 10 s for 15 min after the addition of 2.5 mM *t*-butyl hydroperoxide (Sigma). One unit GPx activity was defined as the oxidation of 1 μmol of NADPH per minute and was related to the protein content of the cell lysate. Using the slope as determined by the MARS software (BMG Labtech V1.11), GPx activity was calculated using the following equation with 0.00379 μM^−1^ as the NADPH extinction coefficient, slope as Delta A 340/min, and units GPx in micromoles NADPH/min:

$$\begin{array}{lll} \left(\frac{\text{Well volume}}{\text{Sample volume}}\right)\times \left(\frac{\text{Slope}\;}{0.00379\;\mu {\text{M}}^{-1}}\right)=\frac{\text{Units}\;\text{GPx}}{g\;\text{protein}\;\text{extract}} & \; & \end{array}$$

### TrxR1 western blot

Cell lysates were resuspended in 4× NuPAGE^®^ LDS sample buffer (Invitrogen) and resolved on a NuPAGE^®^ Novex 4–12% Bis-Tris mini gel (Invitrogen) at 200 V before transfer at 30 V onto a PVDF membrane (Immobilon-FL, Millipore). The membranes were blocked for 1 h in protein-free T-20 TBS blocking buffer (Thermo) at room temperature. The membranes were probed overnight at 4°C with rabbit anti-TrxR1 antibody at 1:1000 (Santa Cruz) and chicken anti-GAPDH at 1:20,000 (Millipore). The secondary donkey anti-rabbit (Li-Cor) and donkey anti-chicken (Li-Cor) infrared labeled antibodies diluted at 1:10,000 and 1:20,000 in 50% blocking buffer/10 mM PBST/0.1% SDS were used for 2 h at room temperature. Bound antibodies were visualized and quantified using the Odyssey imaging system (Li-Cor).

### Migration and invasion assays

Prior to the assays, 10% serum cultured PC3 cells were labeled with 10 mg/ml DilC12 stain (BD Biosciences) in phenol-red free DMEM/F12 GlutaMAX™ (Invitrogen) media for 1 h at 37°C. In addition, the assays were also performed using cells which were adapted to low 2.5% serum levels for 30 days. After labeling, the cells were resuspended in serum free, phenol-red free media, and loaded on top of an 8 μm BD Matrigel™ Matrix-coated membrane of a BD BioCoat™ Tumor Invasion System (BD Biosciences). Uncoated BD Falcon™ FluoroBlok™ 24-multiwell insert plates were included as migration controls. Media containing 5% serum was added to the bottom chamber to create a chemoattractant gradient between the top and bottom chamber. The Fluoroblok plates were covered with sterile gas permeable membrane (Axygen) and incubated in a 5% CO_2_ and 37°C controlled plate reader for 22–44 h. The number of cells migrating or invading was measured every 5 min (544, 590 nm) and fluorescence units were collected at endpoint and analyzed using the MARS software (BMG Labtech, version 1.11).

### Statistical analysis

Statistical significance was determined using a two-tailed *t*-test, and significance level was set at *p* ≤ 0.05 or 0.005. Standard deviations were shown as error bars. Curve fitting was performed where possible using Graphpad Prism 5.

## Results

### Effect of selenium treatment on cell viability *in vitro*

**Figure 1:**
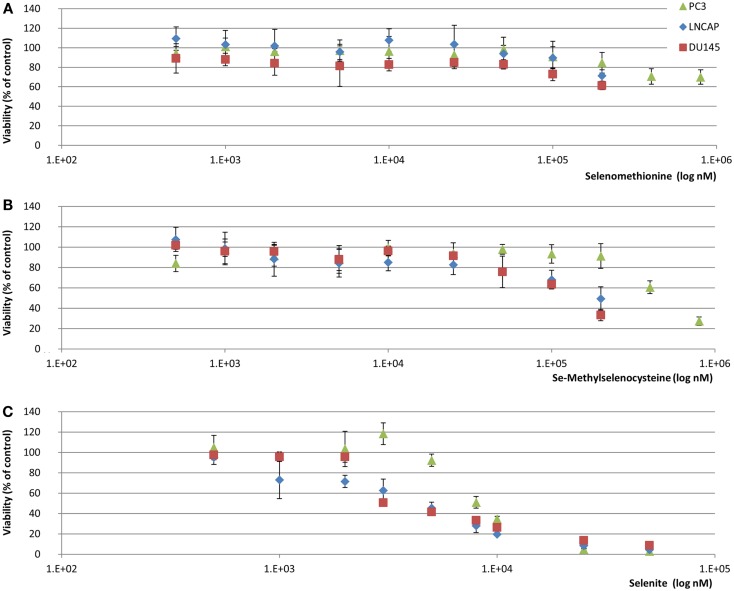
**MTT viability assay of selenium treated prostate cancer cell lines**. Selenium treatment was maintained for 48 h in 10% serum containing conditions and viability was assessed through comparison with metabolic rate of untreated controls (38 nM total selenium from FBS). **(A)** SeMet **(B)** SeMSC **(C)** Selenite. Data represents average of six biological replicates; error bars represent ±SD.

Epidemiological data have demonstrated a narrow margin between selenium deficiency and toxicity. It is therefore important to determine the range of selenium concentrations which correspond to non-toxic, optimal levels *in vitro* systems when investigating the effects of selenium on biological processes. In order to demonstrate minimal toxicity of the selenium compounds and concentrations used, MTT viability analyses of SeMSC (0.5–200 μM), SeMet (0.5–200 μM), and sodium selenite (0.5–50 μM) were performed. The LC50/48 h of SeMSC was recorded and compared to those of SeMet and sodium selenite (Table 1; Figure 1).

**Table 1:** **LC50/48 h concentrations of SeMSC, SeMet, and sodium selenite**.

	SeMet (μM)	SeMSC (μM)	Sodium selenite (μM)
PC3	>800*	606.3†	7.9†
LNCaP	400*	175.6†	4.5†
DU145	300*	141.7†	3.5†

The LC50 values for the various prostate cancer cell lines were determined at 48 h using the MTT viability assay. †Calculated using Graphpad Prism 5 curve fitting.

*Estimate from lower concentrations. LC, lethal concentration.

After treatment with SeMSC we observed a LC50/48 h value of 400–800 μM in PC3, 200 μM in LNCaP, and 150 μM in DU145 cells. Overall, in comparison to the commonly used selenium compounds, SeMSC was found to be 5–10 times less toxic than the sodium selenite but 2 times more toxic than the SeMet. Among the three cell lines studied, PC3 cells were considerably less sensitive to selenium toxicity. These findings are in line with the published literature on selenium toxicity in prostate cancer *in vitro* when considering the different methodologies, compounds, cell lines, and treatment duration used (18–21).

Using identical culture and treatment conditions for all three cell lines, we found that DU145 cells were more sensitive to selenium toxicity than LNCaP and PC3 cells. These data indicate that the concentrations used in the biomarker activity and invasion assays are in the non-toxic range and hence do not adversely affect or interfere with the respective results.

### Effect of selenium treatment on selenium biomarkers *in vitro*

Glutathione peroxidase 1 activity is often used as a biomarker of selenium status and reaches a plateau at plasma selenium concentrations of 1.3 μM (80–120 ng/ml) (27). In cell culture, GPx1 activity is reported to plateau at 30–50 nM selenite and 250–2000 nM SeMet (28). However, various *in vivo* studies have suggested that GPx activity is not a sensitive or reliable biomarker at moderate to high selenium intakes. A rise in serum selenium concentration (from 123 to 196 ng/ml) has been associated with an increase in prostate tissue selenium concentration (1.4–1.6 μg/g) although no effect on GPx activity was observed in prostate tissue (5). These discongruent patterns might have been caused by a high baseline selenium concentration in the study population. Another study published findings that showed a positive correlation between GPx activity in prostate tumor tissue and Gleason score, while an inverse correlation was observed with Selenium binding protein 1 (29). TrxR1 has been associated with increasingly aggressive tumors (30) and has even shown potential as an anti-cancer target (31). As part of a New Zealand prostate cancer selenium trial, the relationship between serum selenium concentration and both TrxR and GPx activity in erythrocytes of 43 participants, aged 50–75 years, with PSA levels above 4 ng/ml, and a negative biopsy was investigated (12). This study reported a positive correlation between baseline serum selenium concentrations (59–128 ng/ml) and both enzyme activities. However, after supplementation (200 or 400 μg/day from selenium-enriched yeast) a 66% increase in serum selenium concentration was reflected in an 80% increase in TrxR activity but not by an increase in GPx activity. This finding suggests that TrxR activity might be a good biomarker at high selenium levels. Hence, we investigated GPx activity and TrxR1 protein expression as selenium biomarkers in LNCaP, PC3, and DU145 cells in response to 48 h or 30 day treatment with SeMSC and SeMet.

#### Effect of SeMSC and SeMet dose-response adaptation on GPx activity under 10% serum culture conditions

As different selenium compounds and culture conditions variably affect the selenium status of the cell lines it is important to note that the blood selenium status and biomarker plateau may not necessarily translate to the appropriate *in vitro* concentration of selenium (32). We measured the GPx activity in LNCaP, PC3, and DU145 cells following 30 day long term exposure and adaptation to SeMSC (500 and 2000 nM) and SeMet (500 nM) to determine the maximum GPx activity and the selenium dose at which this occurs.

**Figure 2:**
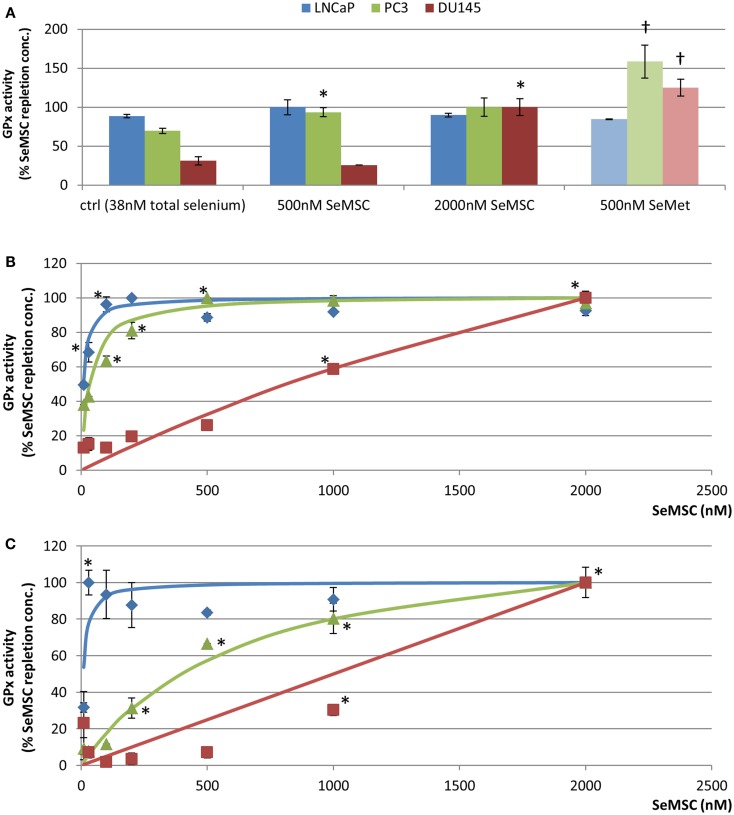
**GPx1 activity in prostate cancer cell lines**. GPx activity was expressed as a percentage compared to the activity at GPx plateau for cells maintained in **(A)** 10% serum after 30 day selenium adaptation to SeMSC or SeMet **(B)** 2.5% serum after 48 h of SeMSC treatment **(C)** 2.5% serum after 30 day selenium adaptation to SeMSC. Data represent average of three biological and three technical replicates, error bars ± SD. *Significant increase compared to previous concentration of SeMSC, †significant increase compared to equal SeMSC concentration *p* < 0.05. Lines represent curve fit using Michaelis–Menten formula [Graphpad Prism 5, *y* = (*V*_max_ × *X*)/(*K*_m_ + *X*)] with **(B)** LNCaP, *V*_max_ = 7.38, *K*_m_ = 9.37, and PC3, *V*_max_ = 2.27, *K*_m_ = 33.77, and **(C)** LNCaP, *V*_max_ = 24.43, *K*_m_ = 8.71, and PC3, *V*_max_ = 4.68, *K*_m_ = 658.8.

Significantly different responses in GPx activity were observed between the three cell lines after exposure to different selenium concentrations (Figure 2A). In LNCaP cells, neither SeMSC nor SeMet induced a significant increase in GPx activity. GPx activity levels were elevated, although not significant, to a maximum of 19.6 ± 1.9 U/g after treatment with 500 nM SeMSC compared to control treatment (17.4 ± 0.4 U/g), whereas SeMet did not affect activity levels. These findings might indicate that LNCaP cells are replete at the selenium concentration (38 nM total selenium) used in the control treatment. In PC3 cells, a significant 1.4-fold increase in GPx activity (*p* = 0.003) was found between the control condition (5.1 ± 0.3 U/g) and 500 nM SeMSC treatment (6.9 ± 0.4 U/g), with a further, although not statistically significant, increase after treatment with 2000 nM SeMSC (7.4 ± 0.9 U/g). Using 500 nM SeMet a significant increase in GPx activity (11.7 ± 1.6 U/g) was observed compared to the control (5.1 ± 0.3 U/g, *p* = 0.002). In DU145 cells, high concentrations of SeMSC, up to 2000 nM, were required to induce a 3.9-fold increase in GPx activity (1.3 ± 0.1 U/g, *p* < 0.001). Even higher SeMSC concentrations may be required to obtain maximum GPx activity as there was a large difference in activity levels between treatment with 500 and 2000 nM SeMSC. Treatment with 500 nM SeMet increased GPx activity to 1.6 ± 0.1 U/g.

Comparison of GPx activity levels between SeMet and SeMSC treatments revealed significant 1.7- and 5.3-fold higher levels at 500 nM SeMet compared to 500 nM SeMSC for PC3 and DU145 cells respectively. The GPx activity at 500 nM SeMet was even higher than that at 2000 nM SeMSC for both cell lines, suggesting that SeMet might have a more profound effect on GPx activity in these cells. A previous study comparing the Gpx inducing potential of selenium compounds has demonstrated a GPx1 plateau in LNCaP cells at 30 nM selenite, at 50 nM selenate, and at 250 nM SeMet (28). In PC3 cells a plateau occurred at 40 nM selenite, at 500 nM selenate, and at 2000 nM SeMet. The authors also observed that GPx1 RNA expression was modulated to a similar extent. Discrepancies between their findings and our data might partly be due to differences in assessment methods (activity assay versus western blot densitometry) and culture conditions, including the culture media and selenium content of the control media. Nevertheless, in accordance to our SeMSC findings they report a higher SeMet induced maximum GPx value in LNCaP cells compared to PC3 cells.

The baseline GPx activity was significantly different between the three cell lines, even when grown under identical conditions. Compared to PC3 and DU145 cells, 3-fold and 40-fold higher GPx activity levels were found in LNCaP cells. The activities under control conditions were 89, 70, and 31% of the maximum GPx activity of SeMSC-treated LNCaP, PC3, and DU145 cells, respectively (Figure 2A), supporting the hypothesis by Rebsch and Colleagues that GPx activity might not be the optimal selenium biomarker for comparison of prostate cancer cell lines at higher selenium concentrations (28). The GPx1 activity values observed in PC3 and DU145 control conditions were similar to those published by Jung et al. who compared the levels of different antioxidant enzymes in LNCaP, PC3, and DU145 cells (33). However, they found LNCaP GPx activity values of about half of those observed in our study which might be explained by differences in cell culture conditions such as the media formulation or the confluency of cells at time of harvesting.

#### Effect of acute and long term adaptation to SeMSC dose on GPx activity under low serum culture conditions

The effect of culture conditions on GPx activity was further explored in cells adapted to low serum conditions (2.5% serum) containing low baseline selenium levels (10 nM total selenium). The impact of both long- (30 days) and short-term (48 h) exposure to SeMSC was investigated to determine GPx activity steady state levels (Figures 2B,C).

Differences were found in the maximum GPx activity levels as well as in the concentration of selenium at which this GPx plateau occurred. LNCaP cells showed the highest GPx activity (7.7 ± 0.02 U/g) with a 2-fold increase compared to the control condition (3.8 ± 0.1 U/g, *p* < 0.001), and reached GPx repletion at a lower SeMSC supplementation concentration (100 nM) compared to the other cell lines. In PC3 cells, the repletion point for GPx activity (2.3 ± 0.0 U/g) was reached at 500 nM SeMSC. The GPx activity of the DU145 control condition was 4.5- and 19-fold lower than those of PC3 and LNCaP cells. The GPx activity significantly (*p* < 0.001) increased between 500 nM (0.4 ± 0.0 U/g), 1000 nM (1.0 ± 0.0 U/g), and 2000 nM SeMSC (1.7 ± 0.1 U/g). Comparing the three cell lines, the GPx activity of the respective control conditions were 87% lower than the maximum activity for DU145, 62% for PC3, and 50% for LNCaP cells. The maximum GPx activities correspond with the *V*_max_ of the Michaelis–Menten curve fit whenever it was possible to calculate these (LNCaP 7.37 ± 0.15 U/g, PC3 2.27 ± 0.08 U/g).

Long term adaptation of cells under low serum conditions resulted in even greater changes in GPx activity in LNCaP cells (Figure 2C). A GPx plateau was reached at 30 nM SeMSC with a >3-fold higher GPx activity (25.7 ± 1.7 U/g) compared to the maximum GPx activity (7.7 ± 0.02 U/g) observed under short-term treatment. Conversely, the maximum GPx activity (1.5 U ± 0.05/g) in DU145 cells was found to be lower than the maximum activity under short-term treatment (1.7 ± 0.1 U/g). In PC3 cells, maximum GPx activity (3.5 ± 0.3 U/g) was obtained at 2000 nM SeMSC which was not statistically different from the activity at 1000 nM SeMSC (2.8 ± 0.3 U/g). Comparing the three cell lines again, the GPx activity of the control conditions were 68–91% lower than their respective repletion point. The maximum observed GPx activities correspond with the *V*_max_ of the Michaelis–Menten curve fit whenever it was possible to calculate these (LNCaP 24.4 ± 0.1.9 U/g, PC3 4.7 ± 0.4 U/g). Longer term adaptation for 30 days to SeMSC treatment under low serum conditions caused lower SeMSC concentration to yield maximum GPx activity in LNCaP cells while higher concentrations were required in PC3 and DU145 cells (Table 2). When comparing 10 and 2.5% serum conditions, it is apparent that the selenium content of serum can obscure an increase in GPx activity after long term selenium treatment. In particular, LNCaP cells show no increase in GPx activity under 10% serum containing conditions while only a small increase could be observed for PC3 cells compared to a pronounced increase in GPx activity under low serum conditions.

**Table 2:** **SeMSC concentrations at which GPx activity plateaus**.

	Time	Serum (%)	SeMSC (nM) at GPx plateau	Max GPx activity (U/g)	*V*_max_ (U/g)
LNCaP	30 days	10	ctrl (38)	19.6	n.a.
	48 h	2.5	100	7.4	7.37 ± 0.15
	30 days	2.5	30	25.7	24.4 ± 0.1.9
PC3	30 days	10	500	7.4	n.a.
	48 h	2.5	500	2.3	2.27 ± 0.08
	30 days	2.5	1000	2.8	4.7 ± 0.4
DU145	30 days	10	≥2000	1.3	n.a.
	48 h	2.5	≥2000	1.7	n.a.
	30 days	2.5	≥2000	1.5	n.a.

Maximum observed GPx activity of LNCaP, PC3, and DU145 cells was assessed using a GPx activity assay and *V*_max_ was calculated using Michaelis–Menten curve fitting in Graphpad Prism 5.

n.a., not available; GPx, glutathione peroxidase.

#### Effect of acute SeMSC treatment on TrxR1 expression under low serum culture conditions

To the best of our knowledge, only one study to date has investigated the effect of selenium treatment on TrxR activity in prostate cancer cell lines. In this work no change in TrxR activity was reported using physiologically relevant doses of selenium in LNCaP and PC3 cells (19). High selenite doses (>5 μM) resulted in a decrease in TrxR activity. However, in the normal prostate BPH-1 cell line an increase in TrxR activity was observed using selenite treatment compared to control. TrxR1 has recently been shown to respond to both selenium and Iberin treatment (34). In accordance, we found that treatment of Caco-2 cells with 25 and 200 nM SeMSC resulted in a 1.4- and 1.5-fold increase of TrxR1 expression respectively, while combination treatment of 200 nM SeMSC with 6 μM Iberin resulted in a 2.1-fold increase (data not shown). Western blotting revealed that a high molecular weight form of TrxR1 was more selenium and Iberin responsive after 48 h treatment than the 55 kDa lower molecular weight form. However, the identity and function of this high molecular weight form remains unknown.

**Figure 3:**
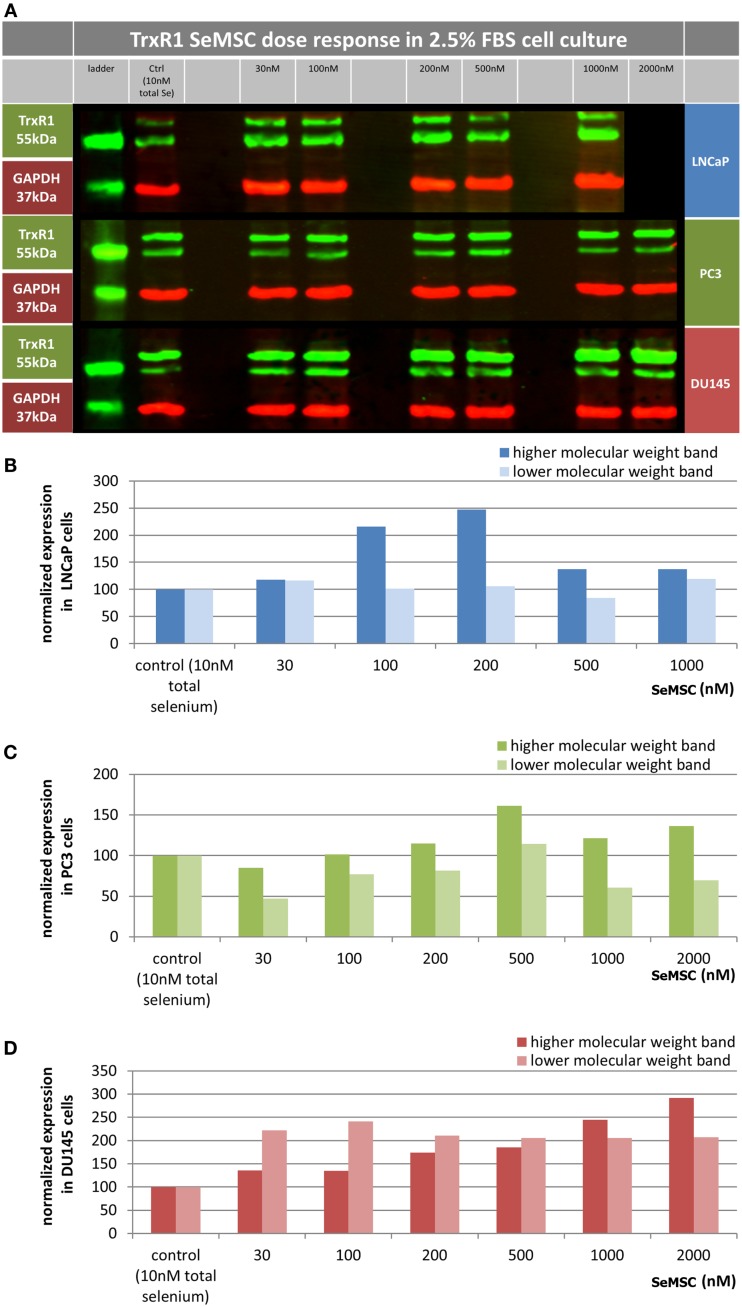
**TrxR1 expression in prostate cancer cell lines**. Cell lines were cultured in either low serum (2.5%) media containing 10 nM of total selenium (control) or media supplemented with 30–2000 nM SeMSC for 48 h. **(A)** Representative image of western blot showing TrxR1 protein bands in green and loading control GAPDH in red. **(B–D)** Quantification of TrxR1 expression normalized to GAPDH and expressed as percentage of control with **(B)** LNCaP, **(C)** PC3, **(D)** DU145, no technical or biological replicates.

We studied the TrxR1 response in DU145, LNCaP, and PC3 cells exposed to SeMSC treatment under low serum conditions (Figure 3). Under 2.5% serum culture conditions, short-term treatment for 24 h of DU145 cells with 100 nM SeMSC induced a 2.1-fold increase in expression, reaching a plateau for the low molecular weight form of TrxR1. Looking at the high molecular weight form, a maximum fold increase of 2.9 was obtained after 24 h treatment with 2000 nM SeMSC, however this cannot be established with certainty as being the plateau point. The high molecular weight TrxR1 was found to be more strongly expressed than the low molecular weight form in both normal and low serum cultured DU145 cells. Low serum culture conditions revealed a maximum expression of the high molecular weight TrxR1 form in LNCaP cells after treatment with 200 nM SeMSC. Surprisingly, this 2.5-fold increase in expression is reduced to 1.4-fold at SeMSC concentrations higher than 200 nM. In contrast with the data obtained from the DU145 cells but in accordance with those from the Caco-2 cells, the lower molecular weight form shows a stronger expression than the higher molecular weight form. In low serum cultured PC3 cells, repletion of TrxR1 expression can be seen for both the high and low molecular weight form after 24 h treatment with 500 nM SeMSC with a 1.6- and 1.1-fold increase respectively. Similar to the LNCaP cells, TrxR1 expression decreased at SeMSC concentrations higher than those of the plateau point. In accordance with the DU145 cells, but not LNCaP and Caco-2 cells, the high molecular weight TrxR1 form shows overall a stronger expression than the low molecular weight form.

When comparing the SeMSC levels required for TrxR1 repletion, we found that the various prostate cancer cell lines respond differently (Table 3). Lower concentrations of selenium were needed for LNCaP cells than for PC3 cells and for PC3 cells than for DU145 cells, which is in agreement with the results of the GPx activity assays. These findings are also in agreement with previously published data on TrxR activity in these cell lines. For instance, PC3 cells have been shown to have a more efficient antioxidant system including a higher TrxR activity than LNCaP cells, while DU145 cells have a slightly higher TrxR activity than PC3 cells (31, 35). It might be of interest to study the TrxR1 selenium responsiveness in long term low serum conditions in more detail. Hereby a TrxR1 activity assay might be a better approach for this purpose since measurement of biomarker activity is functionally more relevant compared to biomarker expression and western blotting is a less accurate method for quantification. Furthermore, it is imperative that the nature and relation of the two TrxR1 forms are explored in future studies.

**Table 3:** **SeMSC concentrations at which TrxR1 expression plateaus as determined by western blotting**.

	Time (h)	Serum (%)	SeMSC (nM) at TrxR1 HMW band repletion
LNCaP	48	2.5	200
PC3	48	2.5	500
DU145	48	2.5	≥2000

HMW, higher molecular weight.

### Effect of SeMSC treatment on the migration and invasion of PC3 cells

**Figure 4:**
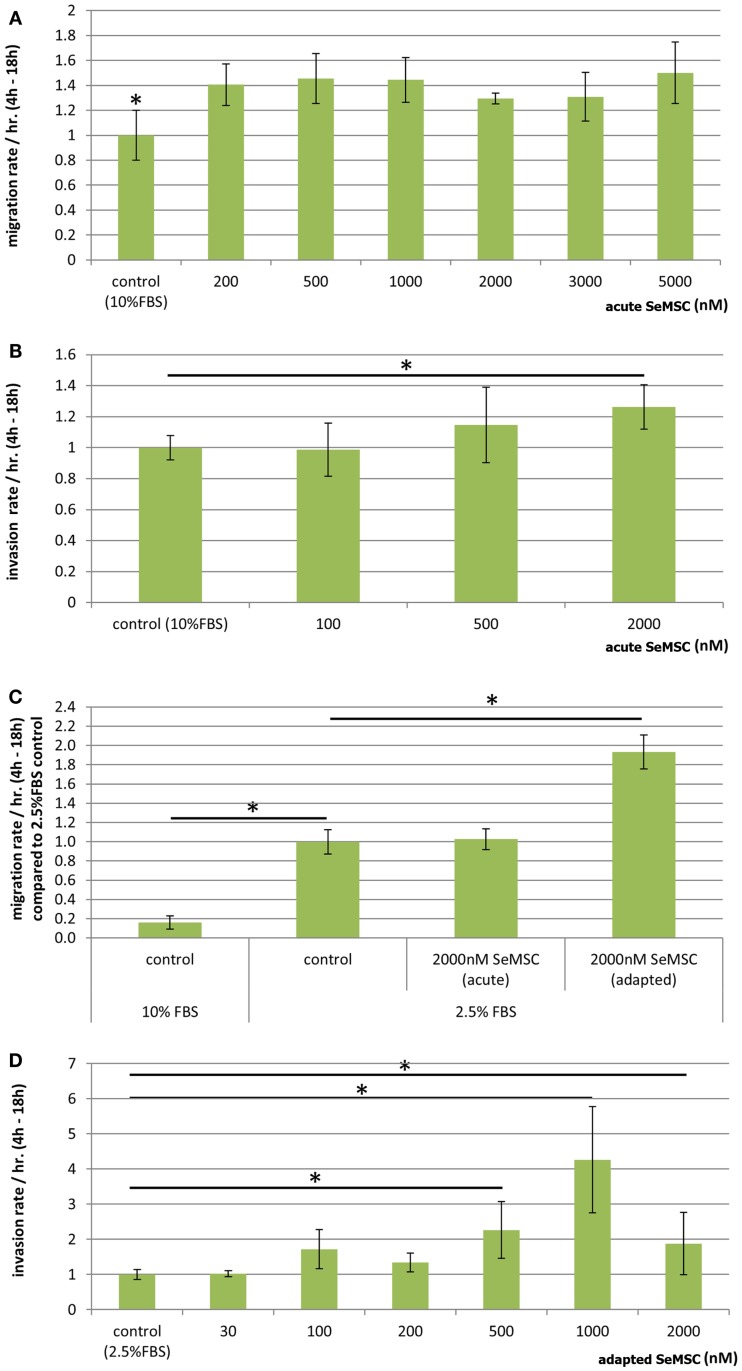
**Migration and invasion of PC3 cells after selenium treatment**. Dose-response study of acute SeMSC treatment on **(A)** migration of PC3 cells cultured in 10% serum containing media **(B)** invasion of PC3 cells cultured in 10% serum containing media, **(C)** migration of 10 and 2.5% serum cultured cells with acute or long term adapted SeMSC treatment, **(D)** 44 h dose-response study of long term/adapted SeMSC treatment on invasion of 2.5% serum cultured cells. Bar charts represent average migration or invasion rate per hour during the linear range of the 24 h assay, three biological replicates, error bars ±SD. *p < 0.01.

The effect of selenium treatment on the migratory and invasive ability of prostate cancer cell lines has not been reported yet. Furthermore, although SeMSC is a dietary relevant form of selenium, no data on its effect on migration or invasion of cancer cells are available. Therefore, we investigated the response of acute or long term SeMSC treatment on both the migratory and invasive behavior of PC3 cells (Figure 4).

We found that long term treatment with SeMSC showed no effect on PC3 migration or invasion through Matrigel, whereas acute treatment for the duration of the assay (22 h) resulted in an increase in migration and invasion. A dose-response study showed a 1.4-fold increase in migration across a range of 200–5000 nM SeMSC (130.4 ± 16.1 FU/h, *p* = 0.002) as compared to the control (93.0 ± 18.6 FU/h) (Figure 4A). On the other hand a clear dose-response increase in invasion was observed (Figure 4B) with 500 nM SeMSC (64.4 ± 13.7 FU/h) and 2000 nM SeMSC (70.9 ± 8.0 FU/h) compared to control cells (56.1 ± 4.4 FU/h).

In addition, both the migration and invasion assays were repeated using low 2.5% serum cultured cells in order to achieve lower baseline selenium concentrations and a greater chemoattractant gradient. In contrast to the results obtained from 10% serum cultured cells, long term treatment under 2.5% serum containing conditions resulted in an increase in migration whereas no difference was found with acute treatment (Figure 4C). More specifically, a significant 1.9-fold increase in migration (2085.1 ± 188.1 FU/h, *p* = 0.0017) was observed with 2000 nM SeMSC long term treatment compared to the control condition (1078.8.1 ± 136.9 FU/h) or 2000 nM acute treatment (1107.9 ± 117.6 FU/h). When investigating invasion, no difference was found for acute treatment whereas long term 2000 nM SeMSC treatment resulted in a 1.8-fold increase as compared to control treatment (data not shown). Therefore, we further investigated the effect of long term SeMSC treatment on PC3 invasion under low serum conditions using a dose-response invasion assay (Figure 4D). A 2.7- and 5-fold increase was found after treatment with 500 nM (239.1 ± 24.3 FU/h, *p* = 0.003) or 1000 nM (440.3 ± 99.9 FU/h, *p* = 0.007) SeMSC respectively. Surprisingly, 2000 nM SeMSC did not further increase invasion but instead reduced invasion back to a 2.4-fold increase (209.0 ± 27.8 FU/h, *p* = 0.006) compared to the control (88.0 ± 12.9 FU/h).

Our data demonstrate an increased migration and invasion of PC3 cells after selenium treatment which are in agreement with publications linking selenoproteins to the invasive potential of macrophages (36). However, several reports have been published showing that selenium has an inhibitory effect on the migration and invasion of cancer cell lines using different forms and doses of selenium. For instance, small amounts of the selenium metabolite methylselenol (<1 μM) have been shown to decrease both HT1080 migration and invasion by 53 and 76%, respectively which might be partly attributed to a decrease in expression of the active form of MMP2. In contrast, no difference in invasion or migration was found after treatment with 5 μM SeMet (14). On the other hand, selenite treatment has been reported to inhibit the invasion of HT1080 sarcoma cells but not their migration or adhesion (13). Further, selenite treatment has been shown to reduce expression of various MMPs in HT1080 sarcoma cells and biopsy derived glioma cells, while no effect on MMP expression was observed in HT1080 cells after selenate treatment. In another study, B16F10 murine melanoma cells treated with selenite showed a decreased migration in a wound healing assay and transwell migration assay. The authors suggest the reduction in migration might be caused by a decreased HIF-1 and VEGF expression, regulated through a decreased IL-18 expression (17).

## Discussion

The present work questions the commonly used practice of using selenium concentrations found in human blood for *in vitro* studies and evaluated the utility of biomarkers such as GPx1 activity and TrxR1 protein expression to determine appropriate selenium levels for *in vitro* work. We studied the use of GPx1 and TrxR1 as biomarkers of SeMSC treatment under different culture conditions and found that GPx activity and TrxR1 protein expression showed a similar selenium repletion pattern and that the effects of selenium treatment on both biomarkers were more pronounced under low 2.5% serum containing conditions. However, we noticed that the maximum levels of GPx1 activity and TrxR1 expression were reached at lower selenium concentrations in LNCaP cells compared to PC3 cells, and in PC3 cells compared to DU145 cells. As such we have been able to demonstrate that one particular physiological relevant dose of selenium, extrapolated from concentrations observed in human blood, can elicit different responses in different prostate cancer cell lines. Therefore using a read-out of selenium repletion such as selenium responsive biomarkers may assist with the selection of which selenium concentration to use in *in vitro* work. Further, we found no relation between the maximum biomarker level and the selenium concentration required to reach that biomarker repletion. For example, LNCaP cells reach the maximum GPx1 activity level at lower selenium concentrations than the other cell lines but their maximum GPx1 activity levels are higher than those of PC3 or DU145 cells. Similarly, maximum protein expression of the high molecular weight TrxR1 form was lower in LNCaP cells compared to PC3 cells, and lower in PC3 cells compared to DU145 cells while lower selenium concentrations were required to result in TrxR1 repletion in LNCaP cells compared to PC3 and DU145 cells.

The effect of SeMSC on the migratory and invasive behavior of PC3 cells was examined under both normal and low serum culture conditions. Selenium treatment induced an increase in migration and invasion in long term low serum conditions. Further studies are required to determine whether the increased invasion is a reflection of increased migration or a combination of increased migration and remodeling of the extracellular matrix. It is hereby important to determine the matrix stiffness as it has been shown that tumor cells can invade extracellular matrices in a protease-independent manner at relatively low matrix stiffness (37). Future work is also needed to investigate which selenium dependent/responsive proteins are involved in this process.

The results of this work indicate that it is essential to determine the selenium sensitivity of an *in vitro* model before investigating the effects of selenium compounds on biological processes, or before comparing models. It also underlines that “golden standard” dose-response curves may not be the best method to determine “physiologically relevant” *in vitro* micronutrient concentrations. It might be more advisable to use appropriate biomarker assessment in future *in vitro* studies.

## Conflict of Interest Statement

The authors declare that the research was conducted in the absence of any commercial or financial relationships that could be construed as a potential conflict of interest.
